# Retrospective analysis of 26 cases of inverted nasal papillomas

**DOI:** 10.1590/S1808-86942012000100004

**Published:** 2015-10-20

**Authors:** Ana Maria Almeida de Sousa, Alcioni Boldrini Vicenti, José Speck Filho, Michel Burihan Cahali

**Affiliations:** a Otorhinolaryngologist, graduate student on health sciences, State Public Servant Medical Care Institute (Instituto de Assistência Médica ao Servidor Público Estadual, IAMSPE)/SP.; b Master's degree in otorhinolaryngology and head & neck surgery, Paulista Medical School, Sao Paulo Federal University. Assistant physician of the Otorhinolaryngology Unit, São Paulo State Public Servant's Hospital, IAMSPE/SP.; c Otorhinolaryngologist and head & neck surgery. Assistant physician of the Otorhinolaryngology Unit, Sao Paulo State Public Servant's Hospital, IAMSPE/SP.; d Doctoral degree in otorhinolaryngology, Medical School, São Paulo University. Assistant physician of the Otorhinolaryngology Unit, São Paulo State Public Servant's Hospital, IAMSPE/SP.

**Keywords:** nose neoplasms, papilloma, inverted, paranasal sinuses

## Abstract

Inverted papilloma (IP) comprises 0.5-4% of benign nasal tumors. The importance is shown by local aggressiveness, a high recurrence rate and the possibility of malignant transformation. The treatment is controversial, but endoscopic approaches tends to be the choice today.

**Aim:**

To describe clinical, epidemiological and treatment of IP cases in a tertiary hospital.

**Methods:**

Retrospective study consisting of chart reviews of 26 patients diagnosed with IP; evaluation of tumor location, clinical staging, follow up, tumor recurrence, malignancy, type of surgery and postoperative complications.

**Results:**

There were 13 men and 13 women, the mean age was 57.8 years. The mean follow up time was 29.4 months; the recurrence rate was 7.6%. There was a preponderance of T3 and T4 tumors and a 3.8% malignancy rate. All patients underwent surgical treatment, mostly endonasal endoscopic surgery.

**Conclusion:**

IP is an uncommon nasal tumor that originates mainly in the lateral nasal wall, but it also affects the paranasal sinuses. Advances in endoscopic surgery are gaining room due to lower invasiveness and success rates similar to traditional external techniques for completely resecting the tumor. There is a lower recurrence rate, and endoscopy a definitive treatment for malignancy cases in this study.

## INTRODUCTION

The inverted papilloma is a benign epithelial tumor of the nasal mucosa and paranasal sinuses in which epithelium invaginates towards the stroma^1^. It originates mostly on the lateral nasal wall or within the maxillary sinus. It affects three males for every female, mostly in the fifth and sixth decades of life^2^. The most common primary sites of the inverted papilloma are the lateral nasal wall (89%), followed by the maxillary sinus (53.9%), the ethmoidal labyrinth (31.6%), the nasal septum (9.9%), the frontal sinus (6.5%), and the sphenoid sinus (3.9%)^3^.

The inverted papilloma comprises about 0.5% to 4% of primary nasal tumors, and it is clinically significant because it is locally aggressive and its recurrence rates range from 5% to 30%. This tumor appears to undergo malignant transformation into squamous cell carcinoma in 5% to 15% cases^4^, ^5^, ^6^, ^7^, ^8^. It may also cause thinning and even erosion of underlying bone, and it may extend into the orbit or intracranial cavity^8^.

It is a rare tumor that has been studied in detail in the past 70 years since its classical description by Ringertz in 1938^9^. The reasons for this are the instigating features of this tumor and the many – controversial – treatment possibilities. Incomplete resection of this tumor is associated with high local recurrence rates; this is matter for concern given the possibility of malignant transformation. Thus, aggressive therapy according to the extension of this tumor is justified^5^, ^9^.

The advent and development of nasal endoscopic surgery, with improved optic angles and magnification power, has made it possible to locate the tumor and its insertion more precisely. Consequently, it is now possible to remove this tumor completely by an endonasal approach, with comparable recurrence rates to aggressive external procedures such as lateral rhinotomy and medial maxillectomy^5^, ^10^.

The purpose of this study was to describe the clinical and epidemiologic aspects and the surgical treatment of inverted papilloma cases diagnosed at the Otorhinolaryngology Unit of the Sao Paulo Public State Servant Hospital in the past 14 years.

## SERIES AND METHOD

The study was a longitudinal historical cohort study – a retrospective clinical study – consisting of data gathering from the registries of patients presenting nasal or paranasal tumors confirmed by pathology as being inverted papillomas, from August 1996 to August 2010.

A standard questionnaire was used for gathering data, focusing on gender, age at diagnosis, prior therapy, biopsy results before surgery, symptoms reported in the first visit, tumor laterality, nasosinusal topography of the tumor, clinical staging, surgical treatment, complications of surgery, treatment of the complications, recurrences, malignancy, and total follow-up time.

Investigation also included a full clinical history, a complete general otorhinolaryngological physical examination, and 3.5 mm flexible fiber nasofibrolaryngoscopy to characterize the tumor. Patients lost to follow-up before surgery, or patients whose registries had insufficient data were excluded from the sample. Computed tomography was done in all patients.

Clinical staging was based on Krouse's^11^ staging system, shown in Chart 1, which is widely used in most studies on this disease.

**Chart 1:** The Krouse staging system^11^.

Staging System for Inverted Papilloma	
T1	Tumor totally confined to the nasal cavity, without extension into the sinuses. The tumor can be localized to one wall or region of the nasal cavity, or can be bulky and extensive within the nasal cavity, but must not extend into the sinuses or into any extranasal compartment. There must be no concurrent malignancy
T2	Tumor involving the ostiomeatal complex, and ethmoid sinuses, and/or the medial portion of the maxillary sinus, with or without involvement of the nasal cavity. There must be no concurrent malignancy
T3	Tumor involving the lateral, inferior, superior, anterior, or posterior walls of the maxillary sinus, the sphenoid sinus, and/or the frontal sinus, with or without involvement of the medial portion of the maxillary sinus, the ethmoid sinuses, or the nasalcavity. There must be no concurrent malignancy
T4	All tumors with any extranasal/extrasinus exension to involve adjacent, contiguous structures such as the orbit, the intracranial compartment, or the pterygomaxillary space. All tumors associated with malignancy

To categorize the treatment, surgery was classified as endonasal endoscopic resection with or without supplementary techniques (sublabial maxillary access), the external approach (lateral rhinotomy, Linch incision, or degloving), and combined approaches.

Recurrences were described as reappearance of tumors after being considered absent (by endoscopy and/ or tomography) in nasal fossae and paranasal sinuses. Residual lesions were remaining tumor mass after surgery, where one or more supplementary treatments were required with not more than six month intervals.

The institutional review board approved this study no. 0135/10.

## RESULTS

The review consisted of 27 registries of patients with a diagnosis of inverted papilloma confirmed by pathology. One patient was excluded after leaving the hospital unauthorized following an incision biopsy for the diagnosis; no treatment was given in this case. No patient was excluded because of insufficient registry data.

Of 26 cases, 13 were female and 13 were male. The mean age at the diagnosis was 57.8 years, ranging from 38 to 78 years. Four (15.3%) patients had already undergone endonasal treatments at other clinics for removing nasal polyps, but these procedures were not well defined. Fifteen patients (57.6%) had an incision biopsy describing the inverted papilloma prior to surgery. The remaining patients were operated for nasal polyps, with the diagnosis of inverted papilloma being as an incidental finding among inflammatory polyps. All incision biopsy results were confirmed after removal of the surgical specimen.

**Graph 1:**
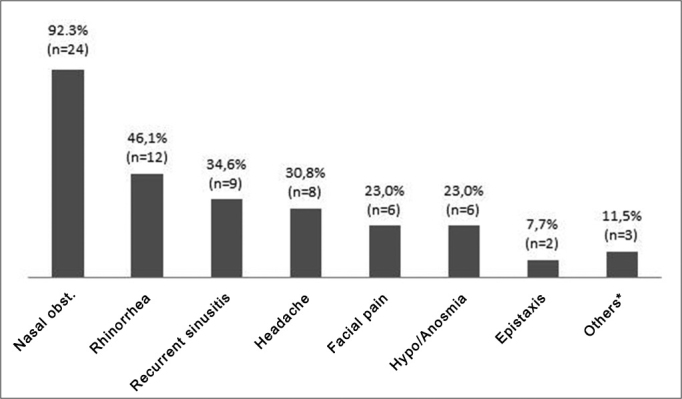
Clinical complaints.

The most frequent complaint was nasal block, in 92.3% of patients. Graph 1 shows this and other complaints. Three patients reported other complaints (others in the Graph), namely hypoacusis, proptosis, and facial edema.

**Graph 2:**
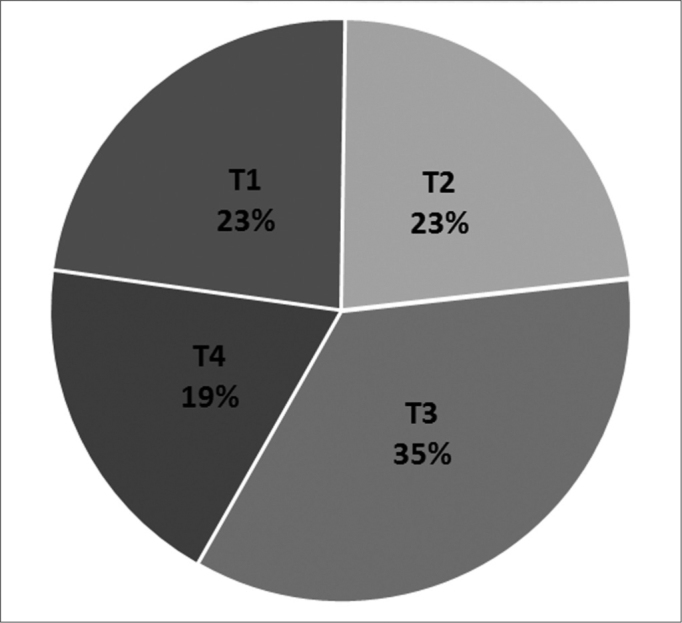
Clinical tumor staging.

The tumor was bilateral in three patients (11.5%), on the left in 10 patients (38.5%), and on the right in 13 patients (50%). The tumor affected the lateral wall in 88.5% of patients, the maxillary sinus in 53.8% of patients, the ethmoid labyrinth in 50% of patients, the sphenoid sinus in 23% of patients, and the frontal sinus in 11.5% of patients. In one case the sphenoid sinus only was involved. The rhinopharynx was affected in 23% of cases. Graph 2 shows the tumor staging (Krouse). In one patient (4%) the tumor degenerated into squamous cell carcinoma.

Endonasal endoscopic resection was performed in 17 patients (65.4%); a supplementary maxillary sublingual approach was done in six of these patients. An external approach only was done in five patients (19.2%), and a combined approach for tumor removal was used in the other four patients (15.4%). There was no relation between tumor staging and the type of surgery or between tumor recurrence or residual tumor and the type of surgery.

The most common postoperative complication, in four patients, was epiphore. One patients developed sinusitis postoperatively and another had transitory dyplopia. Epiphore resolved spontaneously in two patients; one was treated by endoscopic dacryocystorhinostomy and one is about to be treated similarly. Sinusitis resolved by antibiotic therapy.

The mean follow-up time was 29.4 months, ranging from one to 132 months. The tumor recurred in two patients (7.6%), respectively 9 and 60 months after surgery; both had been staged as T3 before the first procedure. Treatment consisted of endonasal endoscopy, which was successful. Five patients had residual tumor after the first procedure. One of these patients had been staged as T2 before surgery; this patient refused further surgery and is being monitored every two months. Two others had been staged as T3, and are waiting for image studies to define further procedures. The remaining two, staged as T3 and T4, were successfully treated to remove the entire tumor, respectively by a sublabial maxillary approach and endonasal endoscopic surgery.

## DISCUSSION

The series at our institution concurred with other papers we reviewed in relation to age of onset of disease – about the fifth and sixth decades of life; the mean age of patients was 57.8 years. This was not the case with gender; the male to female ratio was 1:1 in our sample, whereas it is 3:1 in the literature.

Incision biopsy was done in 57.6% of patients; it confirmed that a diagnosis prior to surgery was useful for surgical planning. Data in the literature have reported the positive predictive value (87.6%), the negative predictive value (94.2%), the sensitivity (78.2%), and the specificity (96.2%) of incision biopsy for the diagnosis of inverted papilloma^12^. This same study showed that tomography and biopsy together are highly accurate for diagnosing this disease. The role of pathology should be emphasized, as a diagnosis was made only after surgery in 42.4% of patients. A diagnosis of inverted papilloma may be made in up to 0.92% of cases of extensive bilateral nasal polyposis^13^. The inverted papilloma may also be found in unilateral polyps with inflammation in the morphological study^14^, which justifies sending all surgical specimens removed by endonasal endoscopic surgery to pathology – a routine procedure at our institution.

The lateral wall was the most prevalent tumor site, which confirms most previously published reports. A few studies have found a higher involvement of the ethmoid sinus compared to the maxillary sinus; others have shown a higher prevalence in the frontal sinus compared to the sphenoid sinus. Our results differ from these reports^3^, ^8^, ^15^. However, because details – especially tomography data – were lacking, it is possible that several cases reported as being on the lateral wall may in fact have been in the ethmoid sinus; this may represent a bias in our study for this datum.

Most cases were staged as T3, which justifies surgery combining endonasal and sublabial approaches to completely resect maxillary sinus tumors. T4-staged tumors were given this classification because in most cases these tumors extended to the rhinopharynx, and in one case malignancy was present. The choice of surgery in each case depended not on the site, but on the extent of tumors and the surgeon's evaluation.

Indications for endoscopic surgery have become more frequent, and larger tumors are now successfully resected with this technique. In recent years we have indicated the external approaches only in exceptional cases – and often combined with endoscopy. The recurrence rate and the presence of residual tumors were similar in a comparison of external approaches and endoscopy; thus, we have safely employed the latter to completely resect these tumors. More extensive endoscopic surgery is possible, such as endoscopic sphenoid nasalization (Soler et al.^16^), which was adopted in one of our patients as a supplementary technique after a residual sphenoid tumor was detected.

It is important to note that recurrences have been found 60 months after the first treatment. Thus, these patients should be monitored to detect recurrences at earlier phases, thereby implying in fewer difficulties when approaching previously operated sites.

## CONCLUSION

The inverted papilloma is a benign nasal neoplasm; it is clinically important because of its local aggressiveness, high recurrence rate, and possibility of malignant transformation.

The clinical data of our series concur with most of the data in our review of the literature.

Although there were Krouse's stage 3 and 4 tumors, endoscopic approaches predominated, even for more extensive tumors. Thus, each surgeon's experience is a determining factor when choosing the type of surgery for these cases.
